# Molecular Hydrogen as a Novel Protective Agent against Pre-Symptomatic Diseases

**DOI:** 10.3390/ijms22137211

**Published:** 2021-07-05

**Authors:** Haru Yamamoto, Yusuke Ichikawa, Shin-ichi Hirano, Bunpei Sato, Yoshiyasu Takefuji, Fumitake Satoh

**Affiliations:** 1 Department of Molecular & Cell Biology, University of California, Berkeley, 3060 Valley Life Sciences Bldg #3140, Berkeley, CA 94720-3140, USA; 2 MiZ Inc., 39899 Balentine Drive Suite 200, Newark, CA 94560, USA; y_ichikawa@e-miz.co.jp; 3 Department of Research and Development, MiZ Company Limited, 2-19-15 Ofuna, Kamakura, Kanagawa 247-0056, Japan; s_hirano@e-miz.co.jp (S.-i.H.); b_sato@e-miz.co.jp (B.S.); f_satoh@e-miz.co.jp (F.S.); 4 Faculty of Environment and Information Studies, Keio University, 5322 Endo, Fujisawa 252-0882, Japan; takefuji@keio.jp; 5 Faculty of Data Science, Musashino University, 3-3-3 Ariake, Koto-Ku, Tokyo 134-8181, Japan

**Keywords:** hydrogen, pre-symptomatic disease, chronic inflammation, oxidative stress, inflammatory diseases, hydroxyl radicals, reactive oxygen species

## Abstract

Mibyou, or pre-symptomatic diseases, refers to state of health in which a disease is slowly developing within the body yet the symptoms are not apparent. Common examples of mibyou in modern medicine include inflammatory diseases that are caused by chronic inflammation. It is known that chronic inflammation is triggered by the uncontrolled release of proinflammatory cytokines by neutrophils and macrophages in the innate immune system. In a recent study, it was shown that molecular hydrogen (H_2_) has the ability to treat chronic inflammation by eliminating hydroxyl radicals (·OH), a mitochondrial reactive oxygen species (ROS). In doing so, H_2_ suppresses oxidative stress, which is implicated in several mechanisms at the root of chronic inflammation, including the activation of NLRP3 inflammasomes. This review explains these mechanisms by which H_2_ can suppress chronic inflammation and studies its applications as a protective agent against different inflammatory diseases in their pre-symptomatic state. While mibyou cannot be detected nor treated by modern medicine, H_2_ is able to suppress the pathogenesis of pre-symptomatic diseases, and thus exhibits prospects as a novel protective agent.

## 1. Introduction

In traditional Chinese medicine, there exists a class of physical conditions known as mibyou, or “pre-symptomatic diseases”, which refers to a state between health and illness where a disease is actively but slowly developing within the body, yet the symptoms are not apparent. A familiar example of mibyou in modern medicine is the array of inflammatory diseases that are commonly referred to as “silent killers”. Pre-symptomatic inflammatory diseases are caused by chronic inflammation, which is characterized by the slow release of proinflammatory cytokines over time in order to induce inflammation in a particular area of the body. Similarly, it is known that the root cause of most pre-symptomatic diseases is chronic inflammation, and one of the major causes of chronic inflammation is known to be excessive oxidative stress on cells, which is impossible to combat with modern medications. As such, finding novel therapeutic methods to treat and prevent the onset of pre-symptomatic diseases, such as inflammatory “silent killers”, has been a great point of emphasis in contemporary medicine.

In a recent publication, we described the potential applications of molecular hydrogen (H_2_) as a therapeutic agent against chronic inflammation in the body [1]. H_2_ is an inert substance in the human body, yet it plays an important role as a selective scavenger of hydroxyl radicals (·OH) in order to prevent the pathogenesis of diseases [2]. ·OH is a reactive oxygen species (ROS) mainly produced in the mitochondria that induces oxidative stress in different cells around the body, which is a key mechanism in the signaling cascade that leads to the development of chronic inflammation. It is known that diatomic H_2_ is the only substance that has the ability to selectively scavenge these ·OH molecules [2]. In addition, given that H_2_ is extremely small in diameter, it has the ability to diffuse in and out of the blood stream, as well as through selective barriers, such as the blood–brain barrier (BBB), to act at various sites in the body [3]. In essence, since H_2_ is able to combat chronic inflammation universally in cells around the body, intaking H_2_, through inhalation or ingestion, may serve as a novel protective agent against mibyou and other pre-symptomatic inflammatory diseases.

Some of the leading causes of death worldwide are inflammatory diseases that develop from mibyou, including various cancers, chronic kidney disease (CKD), diabetes, hepatitis, Parkinson’s disease, Alzheimer’s disease, and hypertension. In contemporary medicine, diseases can be treated once they are diagnosed by identifying characteristic symptoms, using tailored therapeutic methods and medications for specific diseases. This so-called “shotgun therapy” is ineffective against mibyou because symptoms develop during the later stages of the disease when it may be impossible to treat without severe side-effects or the unintentional exacerbation of the symptoms [4]. This review will explore the prospects of H_2_ as a safe, universal, and novel protective agent against common pre-symptomatic inflammatory diseases to corroborate its potential in preventing mibyou. Specifically, the review will outline the mechanisms by which chronic inflammation leads to the pathogenesis of common diseases in their pre-symptomatic state and introduce the ways in which H_2_ can accurately, yet unbiasedly, prevent the onset of such diseases. Mibyou cannot be detected nor treated by modern medicine, and even lifestyle-changing improvements such as exercise have very little effect on the chronic inflammation that underlies such physical conditions. H_2_ can prevent these pre-symptomatic diseases and suppress other intractable diseases that develop from mibyou as a protective agent.

## 2. Chronic Inflammation, the Root Cause of Mibyou and “Silent Killers”

Humans have an extensive immune system by which specialized cells can combat pathogens that enter the body. In particular, inflammation is the process by which macrophages and neutrophils release proinflammatory cytokines, such as interleukin (IL)-1β and IL-18, in direct response to this stimulus [1,5,6,7]. Such a signal is often transient in order to trigger a quick response for the body to fight against these pathogens. However, very occasionally, this signal can be prolonged due to a disturbance in the signaling pathway, leading to a slow yet continuous production of cytokines, known as chronic inflammation. While inflammation generally serves the purpose of preventing infections and diseases, chronic inflammation is a process that, in fact, underlies the pathogenesis of many inflammatory diseases.

Inflammation can be classified into two categories according to the magnitude and duration: acute and chronic inflammation. Common examples of acute inflammation include external injuries and infections, where the symptoms arise suddenly and last a shorter period of time. On the other hand, the prolonged release of cytokines can cause chronic inflammation that develops progressively over a longer span of time. Many forms of mibyou are caused by the latter [2]. By nature, symptoms of chronic inflammation are unnoticeable until the disease has progressed significantly. Nonetheless, the underlying inflammatory processes are active rather than latent, which leads to prolonged damages to internal organs without the patient noticing. Chronic inflammation may eventually cause the dysfunction of specific organs, leading to the onset of common mibyou [2]. From this very fact, inflammatory diseases involving chronic inflammation are often referred to as “silent killers”.

## 3. Molecular Hydrogen Attenuates Chronic Inflammation

### 3.1. Oxidative Stress in Chronic Inflammation

It is extremely important to understand the mechanisms by which the continued release of cytokines occurs in order to stipulate a therapeutic measure that can prevent chronic inflammation in the pathogenesis of diseases. It is known that the root cause of chronic inflammation lies in the activation of inflammasomes, which are multiprotein complexes in the innate immune system that act as sensors of infectious pathogens that enter the human body [8,9,10]. Of particular interest is the nucleotide-binding domain leucine-rich repeat and pyrin domain containing receptor 3 (NLRP3) inflammasome, which is implicated in the development of many known inflammatory diseases and commonly studied in order to inhibit the progression of chronic inflammation [8,9,10]. While the specific mechanism by which NLRP3 inflammasomes are activated is yet to be discovered, it is known that ROS play a key role in the activation step of these innate immune system receptors [1]. More specifically, ROS can oxidize mitochondrial DNA (mtDNA) that bind to NLRP3 inflammasomes in order to activate them [10]. Since the stimulation of NLRP3 inflammasomes lead to the production of cytokines, unregulated activation of these specialized protein complexes may lead to an uncontrolled release of cytokines, resulting in chronic inflammation.

Given that ROS are involved in the activation of NLRP3 inflammasomes, we suggested a mechanism by which oxidative stress in mitochondria can lead to this uncontrolled release of cytokines [1]. Oxidative stress is the process by which the excess production of ROS can lead to damages in the surrounding cells and tissues, including damages to mitochondria. Oxidative stress induced in mitochondria eventually causes mitochondrial dysfunction, which in turn stimulates the uncontrolled production of ROS by the organelle [1]. In the cascade leading to the activation of NLRP3 inflammasomes, this overproduction of ROS can over-oxidize mtDNA, inducing the excess activation of inflammasomes. As a result, targeting the oxidative stress that threatens the normal function of mitochondria may lead to the suppression of NLRP3 activation, and thereby prevent the uncontrolled release of cytokines to regulate chronic inflammation.

### 3.2. Molecular Hydrogen Can Selectively Scavenge Hydroxyl Radicals

While there exist modern treatment methods to control the symptoms of acute inflammation, the same cannot be said for chronic inflammation. Focusing on the cascade that activates NLRP3 inflammasomes gives rise to a novel therapeutic target for many pre-symptomatic inflammatory diseases. This section will focus on the ROS that oxidize mtDNA in order to activate the NLRP3 inflammasome. ROS are mainly produced by mitochondria when there is an imbalance between the production of free radicals and the production of other reactive metabolites [11]. When the activity of excessive ROS produced exceeds that of antioxidant mechanisms in the body, oxidative stress can indiscriminately occur in different cells [11]. Since this excessive stress on cellular organelles eventually leads to dysfunction, consequently triggering signals that lead to the onset of mibyou, it is extremely important to find a therapeutic agent that can selectively eliminate ROS in order to prevent this from occurring.

Amongst the ROS produced in the living body, it is known that ·OH has the strongest oxidizing power, and thus partakes in the most significant damages via oxidative stress [11]. Living cells have enzymes that specifically scavenge other notable ROS, such as superoxide and hydrogen peroxide. That is, superoxide can be scavenged by superoxide dismutase (SOD), and hydrogen peroxide can be scavenged by catalase. However, enzymes that similarly scavenge ·OH have not been found. Conversely, while ·OH induces one of the biggest effects on the health of living cells, there is a single molecule that has been found to be able to eliminate these ·OH before any oxidative stress occurs, which is H_2_.

**Figure 1:**
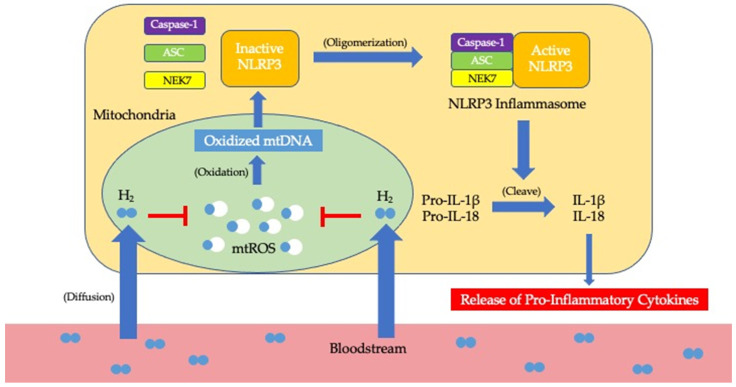
Suggested mechanism by which H_2_ prevents the development of mibyou from chronic inflammation. H_2_ travels around the body in the bloodstream and diffuses into mitochondria of various cells. By eliminating mtROS (e.g., ·OH) produced in mitochrondria, H_2_ prevents the over-oxidation of mtDNA and, consequently, the over-activation of NLRP3. By inhibiting this cascade, H_2_ controls the amount of pro-inflammatory cytokines released, leading to a control over chronic inflammation. H_2_: molecular hydrogen; mtROS: mitochondrial reactive oxygen species; mtDNA: mitrochondrial DNA; NLRP3: nucleotide-binding domain leucin-rich repeat and pyrin domain containing receptor 3; ASC: apoptosis associated speck-like protein containing a CARD; NEK7: NIMA-related kinase 7; IL-1β: interleukin-1β; IL-18: interleukin-18.

It is known that H_2_ plays an important role as a selective scavenger of ·OH that are produced by mitochondria [2]. More specifically, H_2_ is able to reduce ·OH into water molecules via a withdraw reaction that produces no side products nor induces any side effects on surrounding cells [8]. By eliminating excess ·OH, H_2_ inhibits damage to mitochondria via oxidative stress and thereby prevents mitochondrial dysfunction. In doing so, H_2_ controls the amount of ROS released by mitochondria, prevents the over-oxidation of mitochondrial DNA, regulates the activation of NLRP3 inflammasomes, and restrains the overproduction of proinflammatory cytokines (Figure 1). As such, inflammation only occurs at a homeostatic magnitude in response to foreign pathogens in the body. By targeting ROS at the root of chronic inflammation, H_2_ molecules can prevent oxidative stress on mitochondria that may lead to mitochondrial dysfunction and thereby attenuate the risks of developing mibyou via chronic inflammation in the pathogenesis of diseases. This ability for H_2_ to scavenge ·OH brings light to its unique properties as a novel protective agent in preventing chronic inflammation.

### 3.3. Hydrogen Also Affects Mechanisms Other Than ROS Elimination and NLRP3 Activation

It is possible that mechanisms other than oxidative stress by mitochondrial ROS and NLRP3 activation may play a significant role in the development of chronic inflammation. For instance, cellular pathways downstream of pattern recognition receptor (PRR) and toll-like receptor (TLR) activation in the immune system are also known to play a vital role in the development of cellular inflammation. In particular, we discussed the mechanisms of H_2_ in the nuclear factor kappa beta (NF-_K_B) pathway, the mitogen activated protein kinase (MAPK) pathway, and the heme oxygenase 1 (HO-1) pathway as a possible explanation for the anti-inflammatory effects of H_2_ [1]. In the NF-_K_B pathway, it was found that the inhibitory effects of H_2_ on amyloid β (Aβ)-induced neuroinflammation and oxidative stress suppress the activation of NF-_K_B, which is a key transcription factor that regulates the expression of target genes [12]. The MAPK pathway includes many key molecules that are implicated in the transmission of extracellular signals to the nucleus, including the extracellular-signal-regulated protein kinase (ERK), JNK, and p38 MAPK subfamilies. It has been reported that the mechanisms by which H_2_ suppresses inflammation in mice coincides with those that are involved in the modification of signaling in the MAPK pathway [13]. In other experiments, it has been found that the inhalation of H_2_ suppressed lipopolysaccharide (LPS)-induced production of proinflammatory cytokines through the HO-1 pathway [14]. While it has been reported on several occasions that H_2_ indeed has anti-inflammatory effects in the human body, the specific mechanisms by which this occurs have not been determined. As such, it is quite possible that mechanisms other than ROS elimination and NLRP3 activation, which H_2_ has shown to have effects on, may be implicated in the development of chronic inflammation.

## 4. “Machine Gun Therapy” against Mibyou

With modern medicine, it is impossible to detect and treat a disease without recognizing a specific symptom to target, which has limitations against mibyou that are pre-symptomatic. Conversely, H_2_ is able to indiscriminately treat chronic inflammation in various areas around the body without the need to identify the specific disease. In the human body, H_2_ is produced daily by hydrogen-producing gut bacteria, such as Bacteroides and Firmicutes, through a redox process that is catalyzed by the enzyme hydrogenase [15]. Once produced, the permeability and diffusivity of H_2_ allows for the molecule to target ROS produced by mitochondria in various cells throughout the body by diffusing into the bloodstream to be carried around the body [3]. With these properties, H_2_ displays a characteristic “machine gun therapy”, contrary to the “shotgun therapy” that denotes modern medications, which allows for the molecule to target chronic inflammation in various cells around the body [4].

Once H_2_ is dispersed throughout the body, the molecules will target ·OH in cells regardless of whether the ROS is causing oxidative stress or not. This means that in the case of mibyou, the inhalation of H_2_ will reduce oxidative stress in cells and thereby control the release of proinflammatory cytokines to mitigate the risks of chronic inflammation in an unbiased manner. For instance, if inflamed cells existed in both the liver and the kidneys, H_2_ molecules will target the signaling cascade in both locations, and there will never be a case where H_2_ discriminately targets inflammation in one location over the other. H_2_ is multi-targeted by nature [4]. Despite the fact that symptoms of mibyou may not be detected, H_2_ will naturally act as a protective agent against chronic inflammation to prevent the onset of pre-symptomatic inflammatory diseases.

## 5. Hydrogen as a Protective Agent against Inflammatory Diseases

With its role as a selective scavenger of ·OH, H_2_ has broad prospects as a protective agent against inflammatory diseases that arise from mibyou. While the efficacy of H_2_ against diseases in their pre-symptomatic state have not been studied in detail in the past, past clinical trials have in fact showcased this potential. Specifically, studies have been conducted to test the effects of H_2_ on cognitive function in patients diagnosed with mild cognitive impairment (MCI) [16,17]. Notably, Alzheimer’s disease (AD) in its mibyou state is commonly referred to as mild cognitive impairment (MCI), which is a distinctive feature that commonly ensues with aging. Korovljev et al. studied the effects of H_2_ in older women over a span of 4 weeks, during which participants would inhale H_2_ gas for 15 min each day [16]. Comparing the Mini Mental State Exam (MMSE), the Alzheimer disease assessment scale cognitive subscale (ADAS-Cog), and word recall test scores before and after the trial period, study patients showed significant improvement in each of the measurements for cognitive function. In a later study by Nishimaki et al., subjects diagnosed with MCI were treated with either ~300 mL of H_2_ water or placebo water daily to compare the effects of H_2_ in a randomized control study over the span of 1 year [17]. As a result, the ADAS-Cog scores and word recall scores for genotype carriers of apolipoprotein 4 (APOE4) in the H_2_ water group significantly improved in comparison to the control group, confirming that the intake of H_2_ may improve cognitive function. In essence, the two studies showed that H_2_ improved cognitive function in trial participants and corroborated the possibility that H_2_ may indeed serve as a novel protective agent against mibyou [16,17].

The effects of H_2_ against MCI arise from the molecule’s antioxidant properties that prevents oxidative stress in brain cells. Yet, given the knowledge that the molecule is also able to attenuate chronic inflammation, it is possible that H_2_ also acts as a protective agent against other inflammatory diseases in their pre-symptomatic state. As such, this section will explore the mechanisms underlying the pathogenesis of specific inflammatory diseases, namely cancers, CKD, type 2 diabetes, hepatitis, Parkinson’s disease, Alzheimer’s disease, and hypertension, and outline the possibilities by which H_2_ may prevent and treat such diseases in their pre-symptomatic states (Table 1).

### 5.1. Cancers

Despite the development of various treatment methods, such as chemotherapy and radiotherapy, cancer still remains the second leading cause of death worldwide. While mutations and environmental factors are directly tied to the development of various cancers, chronic inflammation in fact underlies many of such mechanisms that cause carcinogenesis [18,19,20,21,22,23,24,25]. It is now known that it is not the proliferation of cancerous cells and tumors alone that causes cancer, but rather the fact that this cell proliferation occurs in environments rich in inflammatory cells and DNA-damage-promoting agents that promote such risks [18]. Tumor cells produce various cytokines that attract leukocytes, such as neutrophils and macrophages, that also produce an assortment of proinflammatory cytokines themselves. In recent years, it has been corroborated that cancer risk, in fact, increases with exposure to various infections, which triggers greater activity by these leukocytes to fight the pathogens and therefore greater production of ROS that induce damage in the DNA of proliferating cells [18]. In a similar fashion, hydrogen peroxide, which is the source of ·OH, can penetrate the nuclear membrane to enter the cell nucleus, and when the radical is generated by the Fenton reaction in the nucleus, DNA strands can be cleaved, causing a genetic mutation [19,20]. Since H_2_ is also able to penetrate the nuclear membrane, it is able to eliminate the ·OH generated in the nucleus to protect against DNA damage induced by ·OH. This DNA damage results in permanent alterations in the form of various genetic mutations that increase risk and promote the further proliferation of tumors in the ‘initiation’ stages of cancer [18]. It has also been reported that major inflammatory pathways involve the activation of transcription factors, such as signal transducer and activator of transcription 3 (STAT3) and NF-_K_B, as well as the activation of NLRP3 inflammasomes, all of which can be mitigated by H_2_ [21,22,23,24,25]. While contemporary treatment methods have served to be relatively effective in decreasing mortality rates of cancer, major limitations remain, including the need for personalized cancer treatments and the onset of severe side effects. In this light, H_2_ acts as a novel therapeutic agent that can prevent carcinogenesis via the elimination of ·OH that induces DNA damage in tumor cells, as well as in inhibiting the activation of NF-_K_B and NLRP3, to reduce chronic inflammation without any side effects.

### 5.2. Chronic Kidney Disease (CKD)

CKD is one of the leading causes of death worldwide, and it is estimated that over 10% of the world’s population is affected by the disease. A characteristic “silent killer”, CKD develops over time with the dysfunction of the kidneys, yet symptoms do not develop until later stages when less than 10% of kidney function is available and the only treatment options are dialysis and kidney transplantation. It is known that the cells that make up the kidneys have more mitochondria than cells that make up other organs because they require more energy to filter blood and thus consume more oxygen in the energy-producing processes [26]. Greater oxygen consumption means that kidney cells face greater risks of oxidative stress. Moreover, the kidneys are an essential organ that are implicated in the release of metabolic waste products, toxins, and pathogens in the blood [27]. Consequently, it is extremely easy for NLRP3 inflammasomes to be activated in the kidneys due a continued exposure to pathogens in the bloodstream that are recognized by macrophages and neutrophils [27]. In recent years, it has been reported that the activation of NLRP3 inflammasomes is directly implicated in the renal release of the proinflammatory cytokines IL-1β and IL-18 and the pyroptosis, or inflammatory cell death, of immune cells in the kidneys [27,28,29,30,31]. In addition, NLRP3 regulates apoptosis in renal tubular epithelial cells by interacting with mitochondria, where NLRP3 mediates the production of ROS to induce mitophagy via oxidative stress [28].

The current standards of therapeutics to combat CKD are such that disease progression is retarded by targeting multiple pathogenic pathways, yet there is no optimized treatment that can cure the disease. As such, it is extremely important to find a therapeutic target that may prevent chronic inflammation so as to avoid the dysfunction of kidneys. H_2_ shows strong prospects as a protective agent against CKD. The permeability and diffusivity of the diatomic molecule allows for H_2_ to travel around the body in the bloodstream, allowing it to be filtered into the kidneys and to penetrate mitochondria of kidney cells [3]. By selectively eliminating ·OH produced by renal mitochondria, H_2_ prevents renal mitophagy, thereby regulating the activation of NLRP3 inflammasomes by ROS, and controlling the release of proinflammatory cytokines. As such, targeting NLRP3 at the root of chronic inflammation with H_2_ presents a novel therapeutic target to prevent kidney dysfunction and therefore the onset of chronic kidney disease.

### 5.3. Type 2 Diabetes

More than 450 million people worldwide live with diabetes, of which approximately 90% have type 2 diabetes that develops over time. It is estimated that over 600 million people will have type 2 diabetes by 2035, and the risks of the disease increase with age, genetic/epigenetic pre-dispositions, overnutrition, and physical inactivity. Type 2 diabetes occurs with a defect in the ability for pancreatic β-cells to secrete sufficient insulin in order to eliminate glucose in the bloodstream. As a result, blood sugar levels slowly increase, leading to the development of diabetes. Several mechanisms have been associated with this decrease in insulin production, many of which are involved in inflammatory processes [32,33]. It is known that elevated blood glucose levels trigger metabolic activity by islet cells, which consequently leads to greater production of ROS by mitochondria [32]. The production of ROS promotes the activation of NLRP3 inflammasomes and capsase-1, which is also implicated in the NLRP3 pathway, consequently leading to the increased release of IL-1β [34,35,36,37]. In addition, it is also known that LPS bound to fetuin-A in the blood stream also increases activation of TLR2 and TLR4, which leads to the translocation of NF-kB and the production of proinflammatory cytokines [34]. It is known that H_2_ plays a key role in both of these mechanisms as a selective scavenger of ROS and in the prevention of the NF-kB pathway. Current therapeutic methods against diabetes encompass a vast array of treatments, including regular exercise and diet restrictions for the maintenance of health to the injection of insulin into the blood stream in order to decrease blood sugar levels. Targeting the inflammatory pathways with H_2_ provides a novel treatment method that requires only the inhalation of H_2_ gas in order to mitigate the risks of type 2 diabetes.

### 5.4. Hepatitis

Hepatitis refers to the severe inflammation of the liver and can be either acute or chronic. There are three main types of hepatitis that are caused by different viruses: hepatitis A caused by the hepatitis A virus (HAV), hepatitis B caused by the hepatitis B virus (HBV), and hepatitis C caused by the hepatitis C virus (HCV). It is estimated that around 325 million people live with hepatitis B and/or hepatitis C, the two most common forms, yet it is extremely rare for symptoms to be apparent in order to diagnose the disease. Vaccines exist for HAV and HBV in populations that are particularly susceptible to the disease, while there is none for HCV. Conversely, therapeutic measures exist for the treatment of hepatitis C, while none exist for both acute and chronic variants of hepatitis A and B. As such, there is a severe lack of a universal treatment method and preventative measure in order to combat the disease.

In recent studies, mice models with constitutive NLRP3 activation have shown higher levels of severe liver inflammation than those without this mutation [38,39]. In addition, it is known that NLRP3 activation results in hepatocyte pyroptosis and liver inflammation, which gives rise to a novel therapeutic target in the treatment of inflammatory liver diseases [40]. Indeed, NLRP3-selective inhibitors such as MCC950 and NR1D1 have shown to reduce inflammation levels in these mice models, consequently preventing hepatitis in the liver [38,39]. Given the ability for H_2_ to regulate NLRP3 activation, it is easy to imagine that the inhalation of the inert gas may act as a protective agent against hepatitis by inhibiting chronic inflammation in the liver.

### 5.5. Alzheimer’s Disease

Approximately 44 million people live with Alzheimer’s disease (AD), which is the most common neurodegenerative disease worldwide. Caused by the activation of microglial cells by the amyloid β (Aβ) peptide, the main cause of AD is known to be cerebral neuroinflammation that leads to symptoms such as memory loss, confusion, and difficulties with dictation [41]. Many studies have shown that increased IL-1β levels are associated with Aβ deposition, which is controlled by the activity of capsase-1 and the NLRP3 inflammasome [41,42,43,44,45,46,47]. In fact, a mice model of AD has shown that decreased NLRP3 inflammasome activity was in fact associated with decreased deposition of Aβ, and therefore a lower risk of AD [42]. AD is referred to as a mild cognitive impairment (MCI) in its pre-symptomatic stages before any characteristic symptoms develop to diagnose the disease as such. In past clinical studies, H_2_ has in fact been shown to treat MCI, and thus, the inert gas presents novel prospects as a protective agent against AD [16,17].

Most contemporary treatment methods against AD and MCI aim to decrease dementia and other cognitive impairment symptoms through medications and management strategies. Since AD is caused by neurodegeneration, an irreversible process that occurs in the nerve cells, there are no treatment methods that may completely cure the disease. However, most approved treatment methods focus on mitigating the symptoms of AD rather than resolving the causative factors at the root of the disease. In fact, the drug aducanumab, or Aduhelm, recently approved by the FDA was the first drug in nearly a decade that aims to decrease levels of Aβ in AD patients. Yet, there are many criticisms against the drug arguing whether the slower cognitive decline outweighs the potential swelling and bleeding of the brain that occurs as a side effect of the drug. In light of these limitations, H_2_ showcases novel prospects as a protective agent against AD. With its ability to penetrate the BBB, H_2_ can eliminate excess ROS that are produced in the brain to prevent the activation of NLRP3 inflammasomes, and therefore decrease the deposition of Aβ, similar to the effects of Aduhelm. Moreover, it is known that H_2_ is an inherently safe substance in the human body that produces no side effects when it scavenges ·OH in the brain [11]. As such, even in comparison to the Aduhelm drug that was recently approved by the FDA, H_2_ may serve as a novel protective agent against AD that prevents the pathogenesis of the disease by targeting the chronic inflammation that may result from Aβ deposition with no side effects to the patient themselves.

### 5.6. Parkinson’s Disease

Parkinson’s disease (PD) is the second most common neurodegenerative disease and affects more than 10 million people worldwide. It is characterized by the loss of dopaminergic neurons in the substantia nigra of the midbrain, which consequently leads to a deficiency in dopamine levels and symptoms such as tremors, movement disabilities, and difficulty talking. While the direct causes of the disease are yet to be discovered, recent studies have shown that a deficiency in components of the NLRP3 inflammasome that activates microglia cells and consequently IL-1β production in the brain leads to greater risk of PD [48,49,50,51,52,53,54]. In other words, NLRP3 inflammasomes and their components are highly expressed in the microglia cells of patients with PD and regulated by the protein α-synuclein and mitochondrial ROS [48]. Additionally, it is known that mitophagy induced by oxidative stress in dopaminergic neurons also plays a major role in the pathogenesis of PD, as mitochondrial abnormalities and dysfunction lead to a great decline in energy production, uncontrolled generation of ROS, and consequently apoptosis of mitochondria in dopaminergic neurons [55]. Similar to AD, PD is also caused by neurodegeneration, and thus there are no cures for the disease. With the importance of preventing the disease in mind, H_2_ serves as a novel protective agent that can regulate NLRP3 activation, as well as a selective scavenger in order to prevent oxidative stress in dopaminergic neurons. Since H_2_ can penetrate the BBB, unlike most contemporary cerebral drugs, the inert gas has great prospects in the prevention of PD.

### 5.7. Hypertension

It is estimated that over 1.13 billion people worldwide have hypertension, defined as having a systolic blood pressure higher than 140 mmHg and a diastolic blood pressure higher than 90 mmHg. Yet, as a characteristic “silent killer,” it is common for people to disregard its presence due to the minimal symptoms that are apparent. In humans, blood pressure is controlled by the renin–angiotensin–aldosterone system (RAAS) and the sympathetic nervous system via the release of blood-pressure-regulating hormones [56]. Any abnormalities to the homeostatic functions of these systems will disrupt the release of hormones and, consequently the regulation of blood pressure. It has been reported that high salt intake induces NLRP3-mediated inflammation in the brain, where the hypothalamus that controls the RAAS and sympathetic system is located [56,57,58]. As a result, neuroinflammation leads to sympathoexcitation and RAAS activation where hormones are over-produced, and thus blood pressure increases [56]. One of the characteristic properties of H_2_ is that it can pass through the BBB due to the molecule’s small diameter. As such, inhaled H_2_ may travel to the brain via the blood stream to prevent neuroinflammation and, therefore, regulate both the RAAS and sympathetic system in order to prevent hypertension.

## 6. Summary

Mibyou refers to a state of disease between health and illness, where a disease is actively but slowly developing within the body yet the symptoms are not apparent. H_2_ has many unique properties that allow for the molecule to be a novel protective agent against mibyou by preventing chronic inflammation that underlies its pathogenesis. This review studied the mechanisms by which common pre-symptomatic inflammatory diseases develop with respect to chronic inflammation and highlighted the possible role of H_2_ in preventing the onset of each of these diseases. In particular, many pathophysiological diseases involve NLRP3 inflammasomes in its development, which acts as a key therapeutic target for H_2_ in preventing these diseases. The permeability and diffusivity of H_2_ allows for the molecule to travel around the body in the bloodstream and inhibit oxidative stress by eliminating ROS produced by mitochondria in various cells. As a result, it is able to prevent chronic inflammation in multiple areas around the body after its production.

It is important to remember that if both CKD and hepatitis were to be developing within a patient, it is impossible for H_2_ to prevent inflammation in the kidneys without doing the same in the liver. That is, the key property that distinguishes H_2_ from modern therapeutic measures is the indiscriminate nature and ability for the molecule to act as a protective agent against mibyou in various areas of the body without the intention of it doing so. This means that even if symptoms of pre-symptomatic diseases are not apparent, the simple act of inhaling H_2_ could prevent all mibyou before common treatment methods need to be applied. Limitations exist today with modern medications and treatment methods against pre-symptomatic diseases. Even exercise, which is essential in the maintenance of human health, cannot suppress the chronic inflammation at the root of such diseases. As such, H_2_ paves the way as a novel preventative agent that can be used universally to avoid the development of chronic inflammation, and more broadly, mibyou.

## Figures and Tables

**Table 1:** Mechanisms of H_2_ in preventing inflammatory diseases that arise from mibyou and chronic inflammation.

Pre-Symptomatic Diseases	Effects or Possible Effects of H_2_	Ref. Nos.
Cancer	Penetrates nuclear membrane to eliminate ·OH produced in the nucleus of tumors and prevents DNA damage. Also prevents activation of STAT3, NF-_K_B, and NLRP3 inflammasome.	[18,19,20,21,22,23,24,25]
Chronic kidney disease	Controls the activation of NLRP3 inflammasomes to prevent chronic inflammation in the kidneys.	[26,27,28,29,30,31]
Type 2 diabetes	Inhibits the activation of NLRP3 and caspase-1 in the NLRP3 inflammasome pathway. Also prevents the translocation of NF-_K_B caused by the activation of TLR2 and TLR4.	[32,33,34,35,36,37]
Hepatitis	Controls NLRP3 activation in liver cells to prevent chronic inflammation in the liver.	[38,39,40]
Alzheimer’s disease (mild cognitive impairment)	Controls cerebral neuroinflammation caused by the activation of microglial cells by Aβ peptide by controlling NLRP3 activation. MCI reduced by preventing oxidative stress in the brain.	[16,17,41,42,43,44,45,46,47]
Parkinson’s disease	Prevents mitophagy and activation of microglia cells by controlling the activation of NLRP3 inflammasomes in dopaminergic neurons	[48,49,50,51,52,53,54,55]
Hypertension	Regulates the RAAS and sympathetic system by penetrating the BBB to attenuate NLRP3-mediated inflammation.	[56,57,58]
